# Qifuyin improves physiological frailty by regulating the intestinal flora in 3xTg-AD mice

**DOI:** 10.3389/fmicb.2026.1753643

**Published:** 2026-04-01

**Authors:** Tianhao Yu, Ying Yu, Junqi Zhao, He Li, Hui Lu, Yangyi Li, Yuqi Peng, Shixue Wang, Wendi Wei, Xiaorui Cheng

**Affiliations:** Innovation Research Institute of Traditional Chinese Medicine, Shandong University of Traditional Chinese Medicine, Jinan, China

**Keywords:** 16S rRNA sequencing, Alzheimer’s disease, dysbiosis, metagenome, physiological frailty, Qifuyin

## Abstract

**Objective:**

Alzheimer’s disease (AD) is often accompanied by motor dysfunction, impaired limb strength, and gut microbiota disturbances. This study aimed to evaluate the effects of Qifuyin (QFY), a traditional Chinese medicine formula, on motor deficits, limb strength, aging, and gut microbiota composition in 3xTg-AD mice, a widely used model of AD.

**Methods:**

Male and female 3xTg-AD mice were administered QFY at low, medium, or high doses. Motor function was assessed using grip strength and rotarod tests. Aging was evaluated through aging scores. Gut microbiota composition was analyzed at the phylum, family, genus, and species levels. Functional profiling of microbiota was performed using KEGG, eggNOG, and carbohydrate-active enzyme (CAZyme) databases. Pearson correlation analyses were conducted to explore relationships between microbiota composition and motor performance.

**Results:**

QFY treatment significantly improved both absolute and normalized grip strength in male and female 3xTg-AD mice. Similarly, motor coordination, as assessed by latency to fall on the rotarod, was significantly enhanced in the groups of QFY. Aging scores were significantly reduced after the treatment of QFY. Microbiome analysis revealed that QFY treatment restored species diversity and improved the overall composition of gut microbiota, with significant increases in Muribaculaceae and decreases in Alcaligenaceae, Rhodanobacteraceae, and Spirochaetaceae. Principal component analysis (PCA) indicated that the gut microbiota composition of the QFY group resembled that of the control (Con) group. Functional analyses showed that treatment of QFY restored microbial pathways related to metabolism and genetic information processing, with significant correlations between microbial alterations and improved motor outcomes. Additionally, QFY modulated the abundance of key carbohydrate-active enzymes, including GH43 and GH35, which were positively correlated with grip strength and rotarod performance.

**Conclusion:**

Qifuyin improves motor function, reduces aging-related deficits, and restores gut microbiota homeostasis in 3xTg-AD mice. These findings suggest that QFY may offer therapeutic potential for addressing frailty and motor dysfunction in AD, in association with alterations in gut microbiota composition and predicted microbial functions.

## 1 Introduction

AD is a progressively debilitating neurodegenerative disorder and is the leading cause of dementia worldwide, posing significant challenges to public health and socioeconomic systems (Mohapatra et al., 2025; Tiwari et al., 2023). The prevalence and incidence of AD continue to rise due to global aging trends. According to the Global Burden of Disease Study 2021, there were nearly 1.92 million deaths and 52.56 million cases of AD and other dementias among individuals aged 60 and older worldwide (Yu et al., 2025). By 2050, the number of AD cases is projected to quadruple to over 82 million, imposing enormous economic and healthcare burdens globally (Ramadan, 2023; Gareri et al., 2024). The primary pathological features of AD include the accumulation of amyloid-beta (Aβ) plaques and hyperphosphorylated tau protein forming neurofibrillary tangles, leading to neuronal damage and progressive cognitive decline (Long et al., 2023; Wang et al., 2023). Thus, there is an urgent need for effective strategies to slow disease progression and improve patient quality of life.

Beyond cognitive impairment, AD is frequently accompanied by non-cognitive symptoms, including decreased limb strength, impaired motor coordination, and general physical frailty, which severely impact daily living abilities and independence (Andrade-Guerrero et al., 2024; Marogianni et al., 2025). Growing evidence suggests that motor dysfunction may precede cognitive decline and serve as an early biomarker for AD progression (Oveisgharan et al., 2024; Petkus et al., 2025). For instance, reduced gait speed has been strongly correlated with increased Aβ deposition, diminished muscle strength, and balance impairment (Marogianni et al., 2025), while a higher burden of AD biomarkers in motor cortices correlates with poorer dexterity performance (Gupta et al., 2024). The presence of motor symptoms, particularly gait disturbances and slowness, has been shown to correlate with an increased risk of rapid cognitive decline (Shaw et al., 2025). These motor dysfunctions are rooted in core AD pathological changes such as neurodegeneration and neuroinflammation (Fakorede et al., 2025). Therefore, improving limb strength and motor coordination in AD patients, thus alleviating physical frailty, has become an essential aspect of daily living ability in comprehensive AD management.

In recent years, increasing attention has been directed toward the role of the gut microbiota in mediating these non-cognitive manifestations of AD. Gut dysbiosis has been shown to induce peripheral and central inflammation (Nayak et al., 2025), increase intestinal permeability (Deleemans et al., 2021), and influence microglial activation and synaptic function through the gut–brain axis (Chen et al., 2025), thereby affecting not only cognition but also motor regulation (Marasco et al., 2022). In parallel, accumulating evidence supports a gut–muscle axis, in which microbial metabolites such as short-chain fatty acids, bile acids, and tryptophan derivatives regulate skeletal muscle protein synthesis, mitochondrial function, and energy metabolism (Zhou et al., 2023). Disruption of these pathways contributes to sarcopenia, reduced grip strength, impaired motor coordination, and ultimately physical frailty. In AD, neurodegeneration, endocrine imbalance, and microbiota-driven systemic inflammation converge, forming a mechanistic continuum linking gut dysbiosis with both motor dysfunction and frailty (Abdol Samat et al., 2025). These findings suggest that interventions capable of remodeling the gut microbiota may exert beneficial effects on both central nervous system and peripheral motor function in AD.

Currently, pharmacological interventions play an important role in the management of physical frailty, yet their application remains limited due to the complex pathogenesis and frequent coexistence of multiple comorbidities. For example, drugs targeting AD—such as cholinesterase inhibitors (donepezil, rivastigmine, and galantamine), N-methyl-D-aspartate (NMDA) receptor antagonists (memantine) and Aβ antibody (lecanemab and donanemab)—are primarily designed to improve cognitive function. However, these agents mainly provide symptomatic relief and exert limited effects on disease modification (Khalil, 2018; Singh et al., 2016). With respect to physical frailty itself, although certain pharmacological agents have been tested to alleviate muscle weakness and related symptoms, the causal relationships between polypharmacy and frailty severity remain incompletely understood, and no drugs have been specifically approved for the treatment of frailty (Campbell and Szoeke, 2009). In addition, aging-related musculoskeletal deterioration, such as osteoporosis and sarcopenia, can be treated pharmacologically, yet these treatments focus mainly on single-organ pathologies and fail to address the multisystemic nature of physical frailty (Mahindran et al., 2021). Given the multifactorial and systemic complexity of frailty, current pharmacotherapies largely aim to mitigate symptoms or manage complications rather than fundamentally reverse or halt its progression. Therefore, there is an urgent need to develop novel therapeutic strategies capable of targeting the core mechanisms of frailty to achieve more comprehensive and effective interventions.

The Chinese herbal formula Qifuyin (QFY) is composed of Panax ginseng, Rehmannia glutinosa (prepared), Angelica sinensis, stir-baked Atractylodes macrocephala, Ziziphus jujuba var. spinosa, Polygala tenuifolia (processed), and honey-fried Glycyrrhiza uralensis. Clinical studies have reported that in a trial involving 20 patients with mild cognitive impairment (MCI), administration of Qifuyin alone for 8 weeks resulted in significant therapeutic efficacy as assessed by the Mini-Mental State Examination (MMSE) score (Xiao et al., 2011). A 12-week treatment with QFY combined with memantine hydrochloride in 40 Alzheimer’s disease patients produced marked improvements in the Mini-Mental State Examination (MMSE), Alzheimer’s Disease Assessment Scale-Cognitive Subscale (ADAS-Cog), and Activities of Daily Living (ADL) scores, indicating that QFY enhanced the therapeutic efficacy of memantine (Tianchen, 2023). A 1-month treatment with QFY combined with Butylphthalide Soft Capsules produced significant improvements in MMSE and MoCA scores among 47 Alzheimer’s disease patients. These results point to its potential to enhance the therapeutic effect of Butylphthalide (Xiaotona, 2021). A meta-analysis, which incorporated 9 randomized controlled trials (RCTs) involving 697 patients, demonstrated that QFY therapy, either as a monotherapy or as an adjunct to conventional Western medications, resulted in significantly enhanced cognitive function in patients with dementia (Wang et al., 2021). However, despite these encouraging findings, existing evidence is predominantly centered on cognitive outcomes, and the potential effects of QFY on physical frailty and motor dysfunction remain largely unexplored. Although we had found that QFY improved the ability of motor coordination, raised survival rate and prolonged the survival days under cold stress stimulation in aged APP/PS1 transgenic mice (Xiao et al., 2022), whether such effects are reproducible in other AD models, particularly those exhibiting both amyloid and tau pathology, and whether they are linked to modulation of the gut microbiota, remains unknown. In the present study, we specifically focused on physical frailty in Alzheimer’s disease. Although various AD mouse models have been widely used to explore cognitive impairment, frailty as a systemic age-related decline has received far less attention in experimental AD research. The 3xTg-AD mouse model develops both amyloid-β and tau pathology in an age-dependent manner, closely mimicking core neuropathological features of AD. However, physiological frailty *per se* has not been systematically evaluated in this model, and its relationship with gut microbiota remains largely unexplored. We therefore selected 3xTg-AD mice in order to investigate whether QFY could ameliorate AD-related frailty and motor dysfunction, and to determine whether these effects are associated with modulation of the gut microbiota.

## 2 Materials and methods

### 2.1 The preparation of QFY

The QFY dry extract powder was purchased from Lunan Pharmaceutical Group Corporation. The specific preparation method is as follows: weigh out 3.0 kg of ginseng (Panax ginseng C. A. Mey.), 4.50 kg of prepared rehmannia root [Rehmannia glutinosa (Gaertn.) Libosch. ex Fisch. & C. A. Mey.], 4.50 kg of angelica [Angelica sinensis (Oliv.) Diels], 2.50 kg of stir-fried Atractylodes macrocephala (Atractylodes macrocephala Koidz.), 3.0 kg of sour jujube seed [Ziziphus jujuba var. spinosa (Bunge) Hu ex H.F.Chow.], 2.50 kg of processed polygala (Polygala tenuifolia Willd.), and 1.50 kg of honey-fried licorice (Glycyrrhiza uralensis Fisch). First, perform heat reflux on the ginseng with 60% ethanol twice, each for 1.5 h. Filter the mixture, set aside the residue, recover the ethanol from the filtrate, and concentrate it to a relative density of 1.03–1.9 (at 60 °C), and set it aside. Next, extract the volatile oil from angelica and stir-fried Atractylodes macrocephala using water distillation, collect the distilled aqueous solution in a separate container, and set aside the residue. The volatile oil ethanol solution is encapsulated with beta-cyclodextrin, dried, and pulverized for later use. Perform boiling of the residues from the above three herbs along with the remaining four herbs (prepared rehmannia root, etc.) in water twice, each time for 2 h. Filter the mixture and mix the filtrate with the concentrated ginseng solution. Perform concentration of the mixture to a relative density of 1.02–1.06 (at 60 °C) to obtain a clear syrup, let it stand, centrifuge, and then concentrate to a relative density of 1.22–1.28 (at 60 °C) to obtain a dense extract. Dry and pulverize the extract, then mix it with the beta-cyclodextrin encapsulated substance.

### 2.2 Animals and treatment

The 3xTg-AD transgenic mice [strain B6;129-Tg (APPSwe,tauP301L)1Lfa Psen1 tm1Mpm/Mmjax], carrying three mutations associated with familial Alzheimer’s disease (APP Swedish, MAPT P301L, and PSEN1 M146V), were purchased from the Jackson Laboratory. The C57BL/6J mice were purchased from Beijing Huafukang Bioscience Co., Ltd. A total of 111 mice were included in the study, comprising 53 males and 58 females. Both the C57BL/6J and 3xTg-AD transgenic mice were housed at the Experimental Animal Center of Shandong University of Traditional Chinese Medicine until they reached 10.3 months of age. All animals were maintained at a temperature of 23 ± 1 °C under a 12-h light/dark cycle with free access to food and water. Before the experiments, all mice were acclimated to the experimental environment for 6 days. All animal-related experiments have been reviewed and approved by the ethics committee of Shandong University of Traditional Chinese Medicine (Ethics No. SDUTCM202209291). All efforts were taken to minimize the number of animals used and their suffering. The 10.3-month-old C57BL/6J and 3xTg-AD transgenic mice were divided into six groups based on activity level and body weight. Each group consisted of 8–9 mice, and the treatment was administered via oral gavage: Con group (male 10, female 10) is C57BL/6J mice, Model (Mod) group (male 7, female 7) is 3xTg-AD transgenic mice, positive drug group (male 8, female 9) is 3xTg-AD + donepezil (1.0 mg/kg/day) and memantine (2.8 mg/kg/day), QFY low-dose group (male 8, female 10) is 3xTg-AD + QFY (1.06 g/kg/day), QFY medium-dose group (male 7, female 10) is 3xTg-AD + QFY (2.12 g/kg/day), QFY high-dose group (male 7, female 9) is 3xTg-AD + QFY (4.24 g/kg/day), The Con and Mod groups were administered distilled water by gavage for the duration of the study. All mice underwent behavioral tests following 305 days of treatment, and samples were collected for biochemical analysis after 328 days of treatment.

### 2.3 Grip strength test

The grip strength meter was placed horizontally and set to the grip strength measurement mode. Each mouse was gently placed on the grid of the apparatus, allowing it to grasp the mesh firmly with all four limbs. The mouse was then gently pulled backward by the tail in a horizontal direction until its forelimbs and hindlimbs released the grid. At this point, the instrument automatically recorded the maximal grip force. Each mouse was measured three consecutive times with a 30-s interval between trials. The mean value of the three measurements was taken as the grip strength of the mouse. To account for body weight differences, the results were normalized using the following formula: normalized grip strength = Mean grip strength/Body weight.

### 2.4 Rotarod test

Mice were first subjected to adaptive training on a rotarod apparatus. The training program was set to accelerate uniformly to 10 rpm within 20 s and then maintained for 280 s. Six mice were placed simultaneously in the six lanes of the rotarod apparatus. Before the start of training, each mouse was allowed to adapt on the rod for 30 s. After adaptation, the preset program was initiated until the training ended or the mouse fell off. Twenty-four hours after the adaptive training, the formal test was conducted. The program was set to accelerate uniformly to 15 rpm within 45 s and then maintained for 155 s. Six mice were placed simultaneously in the six lanes of the apparatus. Each mouse was allowed to adapt on the rod for 30 s prior to the start of the test. After adaptation, the preset program was initiated. During the test, a trial was considered terminated when the mouse fell off the rod or clung to the rod and rotated passively for three consecutive turns. The apparatus automatically recorded the latency to fall (time spent on the rod). Each mouse underwent three trials with a 30-min inter-trial interval to allow sufficient rest. The average latency across the three trials was taken as the final result, which was further normalized to body weight.

### 2.5 Aging score assessment

The aging assessment was conducted based on the scoring system originally established by Professors Toshio Takeda and Masanori Hosokawa at Kyoto University, combined with our group’s previous research on aging evaluation. A multidimensional aging scale was developed incorporating both behavioral and morphological characteristics of mice. The assessment included four domains: behavioral responses, skin and hair condition, ocular condition, and spinal condition. Each parameter was graded on a five-point scale (1–5), with higher scores indicating more severe aging, and a score of 5 representing the most advanced aging state.

### 2.6 Sample collection

Fecal samples from each mouse were collected directly after spontaneous excretion into sterile EP tubes, sealed with parafilm, immediately snap-frozen in liquid nitrogen, and subsequently stored at −80 °C until further analysis.

### 2.7 16 srRNA

DNA extraction: Approximately 0.25 g of sample was placed into a 2-ml centrifuge tube, followed by the addition of 500 μl Buffer SA and 100 μl Buffer SC. Eight 3-mm grinding beads and 0.2 g of 1-mm grinding beads were then added, and the mixture was homogenized using a TGrinder H24 tissue homogenizer (TIANGEN, OSE-TH-01) under the following conditions: oscillation at 6 m/s for 20 s, with a 10-s interval, for a total of two cycles. The homogenate was then incubated at 70 °C for 15 min for lysis. The lysate was centrifuged at 12,000 rpm (∼13,400 × *g*) for 1 min, and approximately 500 μl of the supernatant was transferred into a new 2-ml centrifuge tube. Subsequently, 200 μl Buffer SH was added, vortexed for 5 s, and incubated at 4 °C for 10 min. After centrifugation at 12,000 rpm for 2 min at room temperature, the vacuum-sealed, pre-packed 96-deep well plate from the kit was mixed by inversion several times to resuspend the magnetic beads. After removing the vacuum package, the plate was gently tapped to collect all reagents and beads at the bottom (alternatively, centrifuged at 500 rpm for 1 min). The aluminum sealing film was carefully removed prior to use to avoid spillage. Automated extraction: The TGuide S96 automated nucleic acid extraction and purification system (TIANGEN) was used to run the soil/fecal genomic DNA extraction program according to the manufacturer’s protocol. DNA quality control: The extracted nucleic acids were eluted in 35–50 μl TB buffer and stored at −20 °C until further use. DNA concentration was measured using a Qubit 3.0 fluorometer (Invitrogen) with the Qubit dsDNA HS Assay Kit, and DNA integrity was evaluated by 1% agarose gel electrophoresis.

### 2.8 Metagenomic sequencing

Library preparation was performed using the VAHTS^®^ Universal Plus DNA Library Prep Kit for Illumina (ND617) according to the manufacturer’s instructions. Library quality was assessed using a Qsep-400 system for fragment analysis, and library concentration was quantified with a Qubit 3.0 fluorometer. Libraries meeting the following criteria were subjected to sequencing: concentration ≥ 1 ng/μl, fragment size distribution with a central peak of 430–530 bp and an average size of 420–580 bp, a normal peak shape, and no detectable secondary peaks. Qualified libraries were sequenced on the Illumina NovaSeq 6000 platform using a paired-end 150 bp (PE150) strategy. Metagenomic sequencing and data processing: metagenomic libraries were constructed using the VAHTS^®^ Universal Plus DNA Library Prep Kit for Illumina (ND617) according to the manufacturer’s protocol. Library quality was evaluated using a Qsep-400 system for fragment size distribution, and library concentration was quantified with a Qubit 3.0 fluorometer. Libraries meeting the following quality control thresholds were selected for sequencing: concentration ≥ 1 ng/μl, fragment size distribution with a central peak of 430–530 bp and an average size of 420–580 bp, unimodal and normally distributed peak shape, and absence of secondary peaks. Qualified libraries were sequenced on the Illumina NovaSeq 6000 platform with a paired-end 150 bp (PE150) strategy. Raw sequencing reads were first subjected to quality control using FastQC and Trimmomatic to remove low-quality bases and adapter sequences. Host-derived sequences were filtered by aligning reads to the mouse reference genome (GRCm39) using Bowtie2, and non-host reads were retained for downstream analysis. High-quality clean reads were assembled into contigs with MEGAHIT, and open reading frames (ORFs) were predicted using Prodigal. Non-redundant gene catalogs were constructed with CD-HIT, and functional annotation was performed against the KEGG, eggNOG, and CAZy databases using DIAMOND. Taxonomic classification was carried out using Kraken2 with the NCBI RefSeq database. Relative abundances of taxa and functional categories were calculated based on mapped read counts.

## 3 Results

### 3.1 QFY enhanced the limb strength of 3xTg-AD mice

**FIGURE 1:**
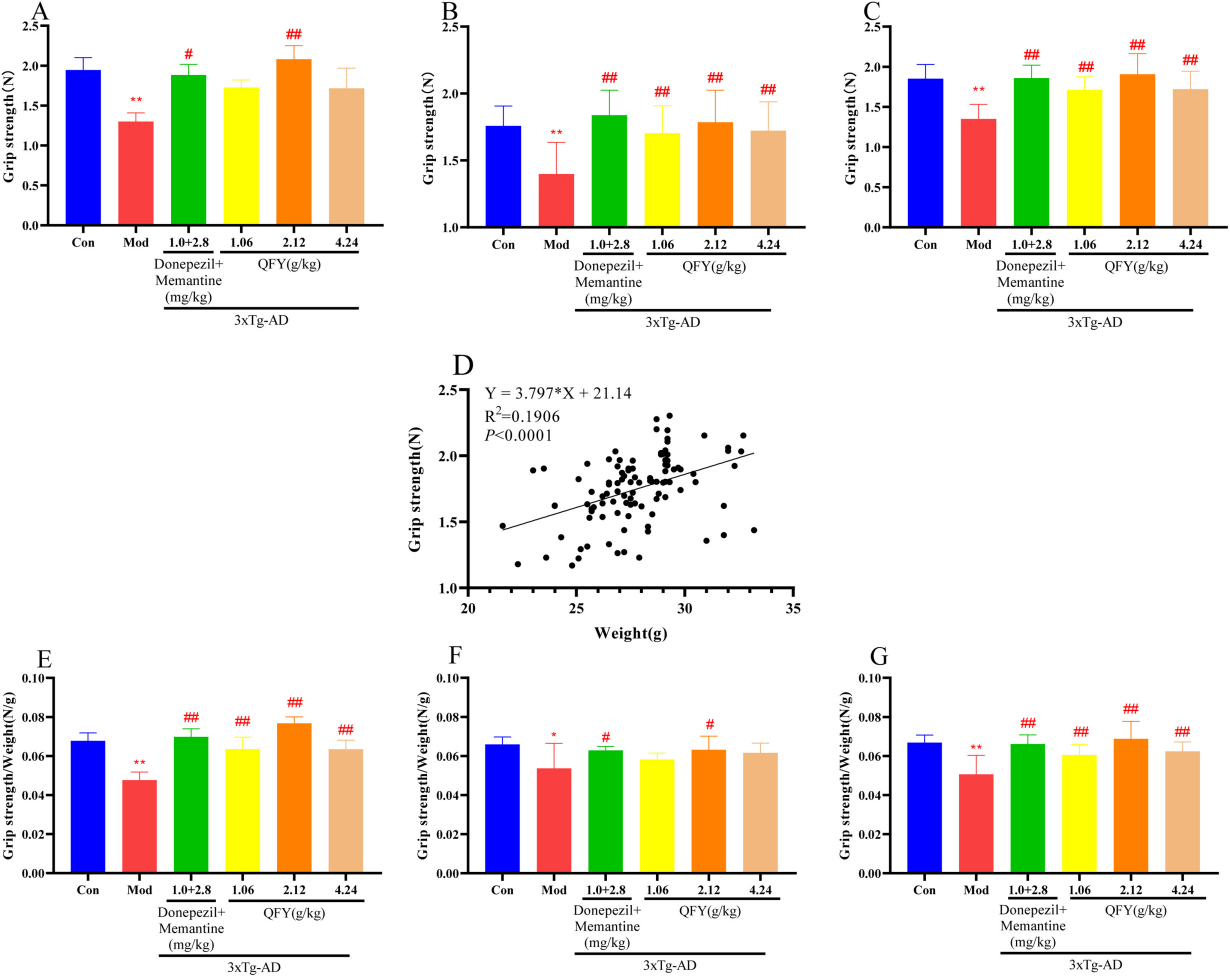
QFY enhanced the limb strength of 3xTg-AD mice. **(A)** Grip strength of male mice. **(B)** Grip strength of female mice. **(C)** Combined grip strength of male and female mice. **(D)** Correlation between grip strength and body weight. **(E)** Grip strength/body weight in male mice. **(F)** Grip strength/body weight in female mice. **(G)** Combined grip strength/body weight in male and female mice. Mean ± SD, *n* = 7–20, *P < 0.05, **P < 0.01 vs. Con, Student‘s *t*-test, ^#^*P* < 0.05, ^##^*P* < 0.01 vs. Mod, One-way ANOVA followed by Dunnett’s multiple comparisons test, Graphpad 8.0.1.

Correlation analysis revealed a positive association between body weight and absolute grip strength (Figure 1D, *P* < 0.0001). The stronger correlation observed for normalized grip strength suggests that the weight-adjusted measure provides a more reliable indicator of neuromuscular function.

In male mice, the Mod group exhibited a significant weaker in absolute grip strength (Figure 1A, *P* < 0.01) and in normalized grip strength (Figure 1E, *P* < 0.01) relative to Con. Compared with the Mod group, the positive drug group showed significant strength in both absolute grip strength (Figure 1A, *P* < 0.05) and normalized grip strength (Figure 1E, *P* < 0.01). The administration of QFY at a low dose (1.06 g/kg) significantly improved normalized grip strength (Figure 1E, *P* < 0.01). The medium dose of QFY (2.12 g/kg) further enhanced both absolute and normalized grip strength (Figures 1A, E, *P* < 0.01), while the high dose (4.24 g/kg) significantly improved normalized grip strength (Figure 1E, *P* < 0.01).

In female mice, the Mod group showed significantly decreased absolute grip strength (Figure 1B, *P* < 0.01) and normalized grip strength (Figure 1F, *P* < 0.05) compared with Con. Relative to the Mod group, the positive drug group significantly increased absolute grip strength (Figure 1B, *P* < 0.01) and normalized grip strength (Figure 1F, *P* < 0.05). Low-dose QFY significantly improved absolute grip strength (Figure 1B, *P* < 0.01). The medium dose significantly enhanced both absolute and normalized grip strength (Figure 1B, *P* < 0.01; Figure 1F, *P* < 0.05), and the high dose significantly enhanced the absolute grip strength (Figure 1B, *P* < 0.01).

When data from male and female mice were combined, the Mod group displayed significantly lower absolute grip strength (Figure 1C, *P* < 0.01) and normalized grip strength (Figure 1G, *P* < 0.01) than the Con group. Compared with the Mod group, the positive drug group significantly improved both absolute grip strength (Figure 1C, *P* < 0.01) and normalized grip strength (Figure 1G, *P* < 0.01). The treatment of QFY at low, medium, and high doses significantly increased both absolute and normalized grip strength (Figures 1C, G, *P* < 0.01). In a comparison of the two sexes, male mice in the medium-dose QFY group had significantly superior grip strength.

### 3.2 QFY improved the motor coordination ability of 3xTg-AD mice

**FIGURE 2:**
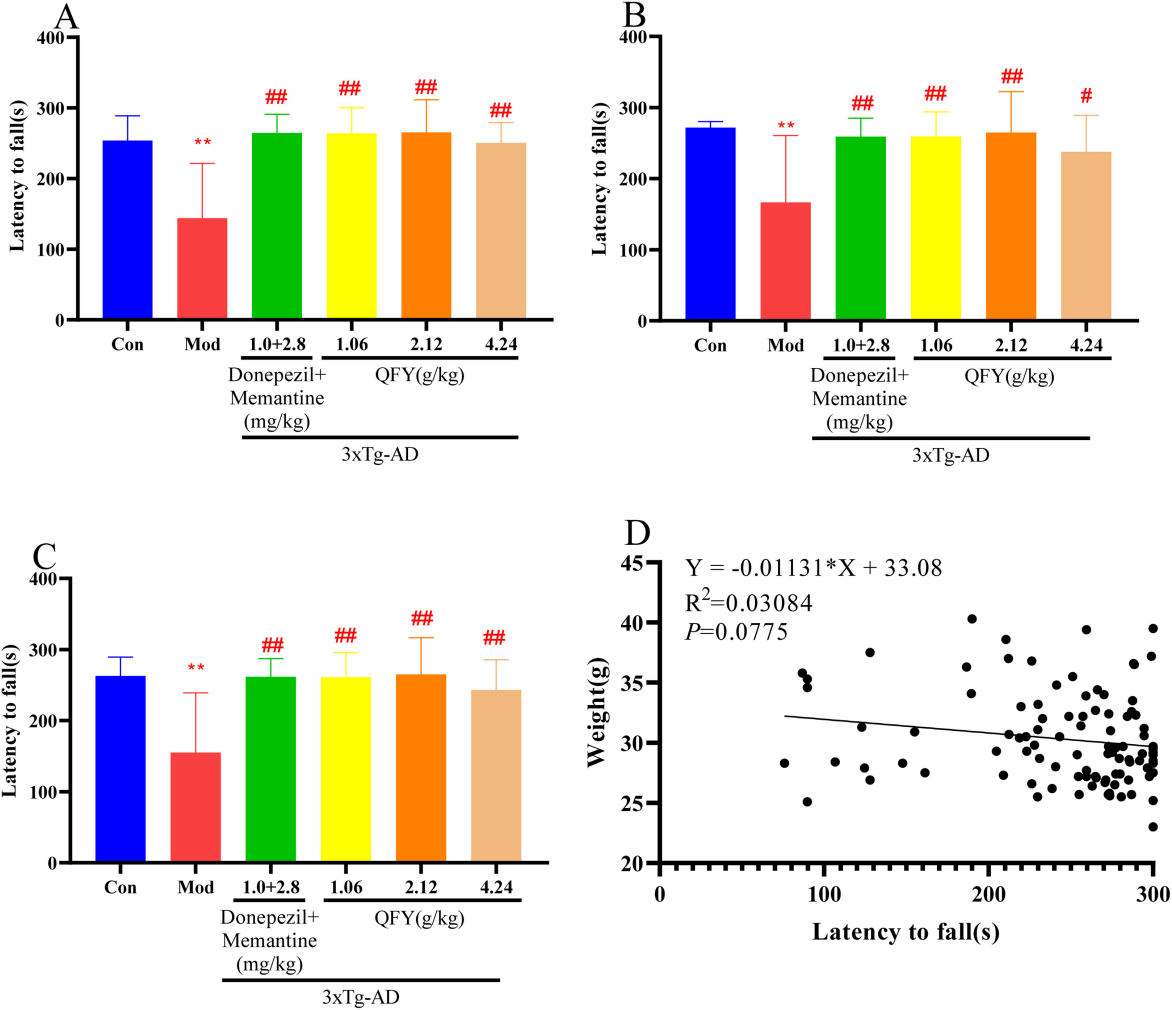
QFY improved the motor coordination ability of 3xTg-AD mice. **(A)** Latency to fall in male mice. **(B)** Latency to fall in female mice. **(C)** Combined rotating time of male and female mice. **(D)** Correlation between latency to fall and body weight. Mean ± SD, *n* = 7–20, **P < 0.01 vs. Con, Student‘s *t*-test, ^#^*P* < 0.05, ^##^*P* < 0.01, vs. Mod, One-way ANOVA followed by Dunnett’s multiple comparisons test, Graphpad 8.0.1.

Correlation analysis revealed no significant relationship between body weight and rotarod performance, as assessed by absolute latency to fall (Figure 2D, *P* > 0.05), indicating that the motor coordination deficits in 3xTg-AD mice were independent of body weight.

In male mice, the Mod group exhibited a significantly shorter the latency to fall on the rotating rod compared to Con (Figure 2A, *P* < 0.01). Compared with the Mod group, the treatment of positive drug significantly prolonged the latency to fall (Figure 2A, *P* < 0.01). Administration of QFY at low, medium, and high doses also significantly increased latency to fall relative to the Mod group (Figure 2A, *P* < 0.01).

In female mice, the 3xTg-AD Mod group showed a significant decrease in latency to fall compared with Con (Figure 2B, *P* < 0.01). The positive drug group demonstrated a significant extension in latency to fall relative to the Mod group (Figure 2B, *P* < 0.01). Both low- and medium-dose QFY significantly increased latency to fall (Figure 2B, *P* < 0.01), while high-dose QFY also resulted in a significant improvement (Figure 2B, *P* < 0.05).

When data from both sexes were combined, 3xTg-AD mice displayed significantly shorter latency to falls than Con (Figure 2C, *P* < 0.01). Compared to the Mod group, the positive drug group showed a significant increase in latency to fall (Figure 2C, *P* < 0.01). Similarly, the treatment of QFY at all tested doses significantly improved latency to fall (Figure 2C, *P* < 0.01).

No significant differences were observed between males and females across all groups.

### 3.3 QFY reduced the aging score of 3xTg-AD mice

**FIGURE 3:**
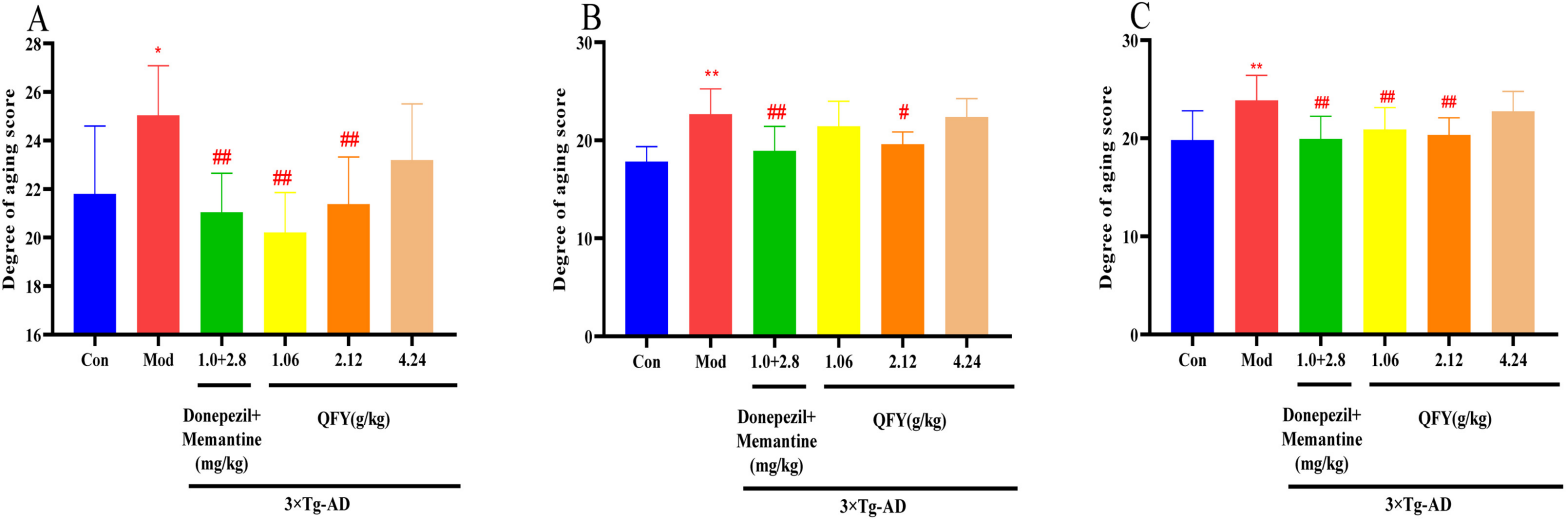
QFY reduced the aging score of 3xTg-AD mice. **(A)** Aging score in male mice. **(B)** Aging score in female mice. **(C)** Combined aging score of male and female mice. Mean ± SD, *n* = 7–20, *P < 0.05, **P < 0.01 vs. Con, Student‘s *t*-test, ^#^*P* < 0.05, ^##^*P* < 0.01 vs. Mod, One-way ANOVA followed by Dunnett’s multiple comparisons test, Graphpad 8.0.1.

In male mice, the 3xTg-AD group exhibited a significantly higher aging degree score compared to Con (Figure 3A, *P* < 0.05). Relative to the Mod group, the positive drug group, the low- and medium-dose QFY groups showed significant reductions in aging degree scores (Figure 3A, *P* < 0.01).

In female mice, aging degree score were also markedly elevated in the 3xTg-AD group relative to Con (Figure 3B, *P* < 0.01). Compared with the Mod group, the treatment of positive drug significantly lowered the aging degree score (Figure 3B, *P* < 0.01). The medium dose resulted in a significant decrease (Figure 3B, *P* < 0.05).

When data from both sexes were pooled, the 3xTg-AD group continued to demonstrate a significantly higher aging degree score than the C57 group (Figure 3C, *P* < 0.01). Compared to the Mod group, the positive drug group showed a significant reduction in aging degree score (Figure 3C, *P* < 0.05). Both low- and medium-dose QFY markedly reduced aging degree scores (Figure 3C, *P* < 0.01). A comparison between the sexes revealed a significantly lower aging score in females compared to males.

### 3.4 The treatment of QFY improved the species diversity of gut microbiota in 3xTg-AD mice

**FIGURE 4:**
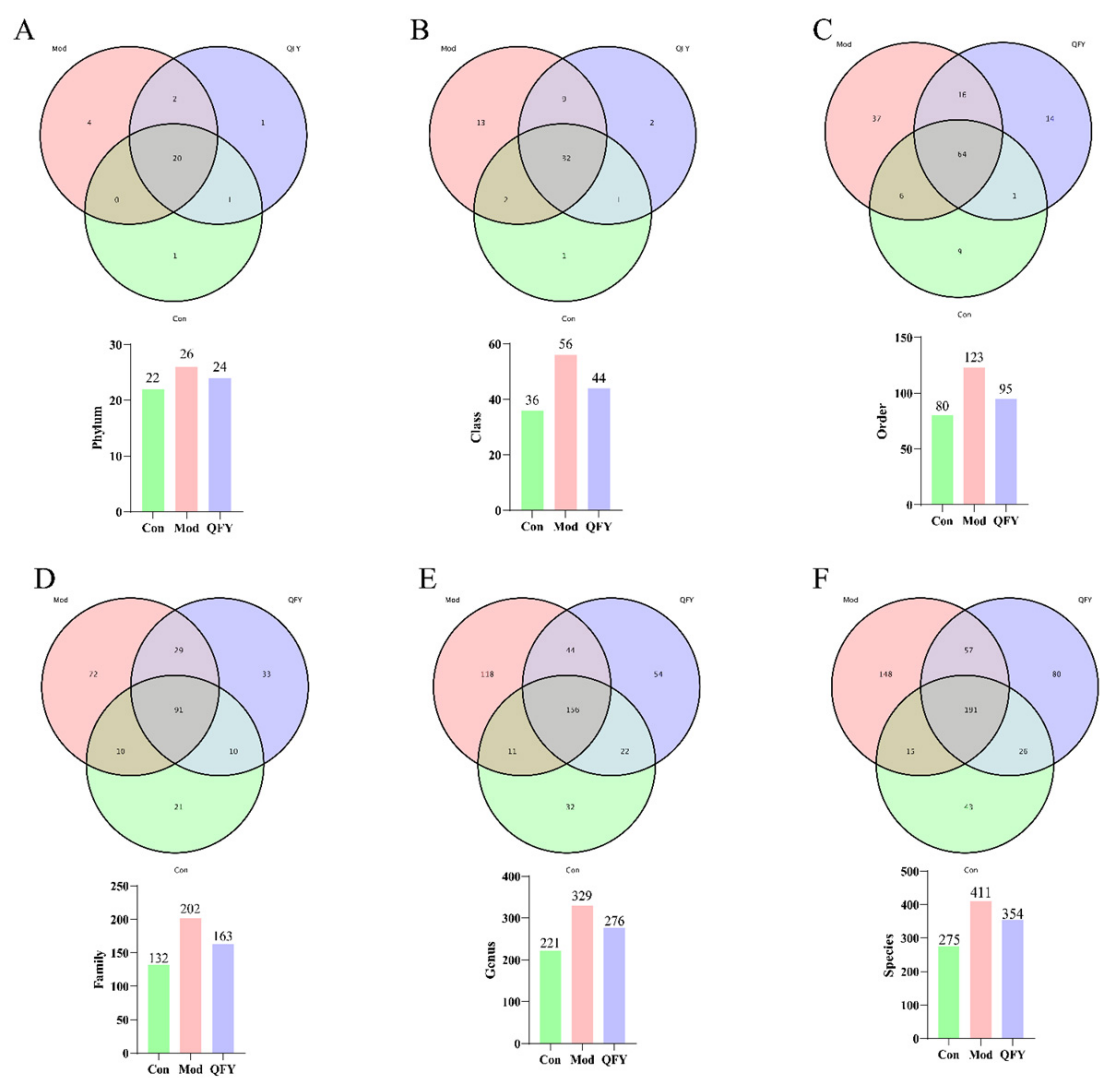
The treatment of QFY improved the species diversity of gut microbiota in 3xTg-AD mice. **(A)** Phylum-level species count. **(B)** Class-level species count. **(C)** Order-level species count. **(D)** Family-level species count. **(E)** Genus-level species count. **(F)** Species-level species count.

At the phylum level, the Con and QFY groups shared one common taxon, whereas the Con and Mod groups shared none (Figure 4A). At the genus level, the Con and QFY groups shared 22 taxa, compared with only 11 taxa shared between the Con and Mod groups (Figure 4E). At the species level, 26 taxa were common to the Con and QFY groups, whereas only 15 taxa were shared between the Con and Mod groups (Figure 4F). We further observed that, at taxonomic levels below the phylum, species richness in 3xTg-AD Mod mice exceeded that of Con; following QFY treatment, species number decreased and became more comparable to that of the Con group (Figures 4A–F). These results indicate that QFY exerts a restorative regulatory effect on the gut microbiota composition of 3xTg-AD mice.

### 3.5 QFY modulates gut microbiota composition and β-diversity at the family level in 3xTg-AD mice

**FIGURE 5:**
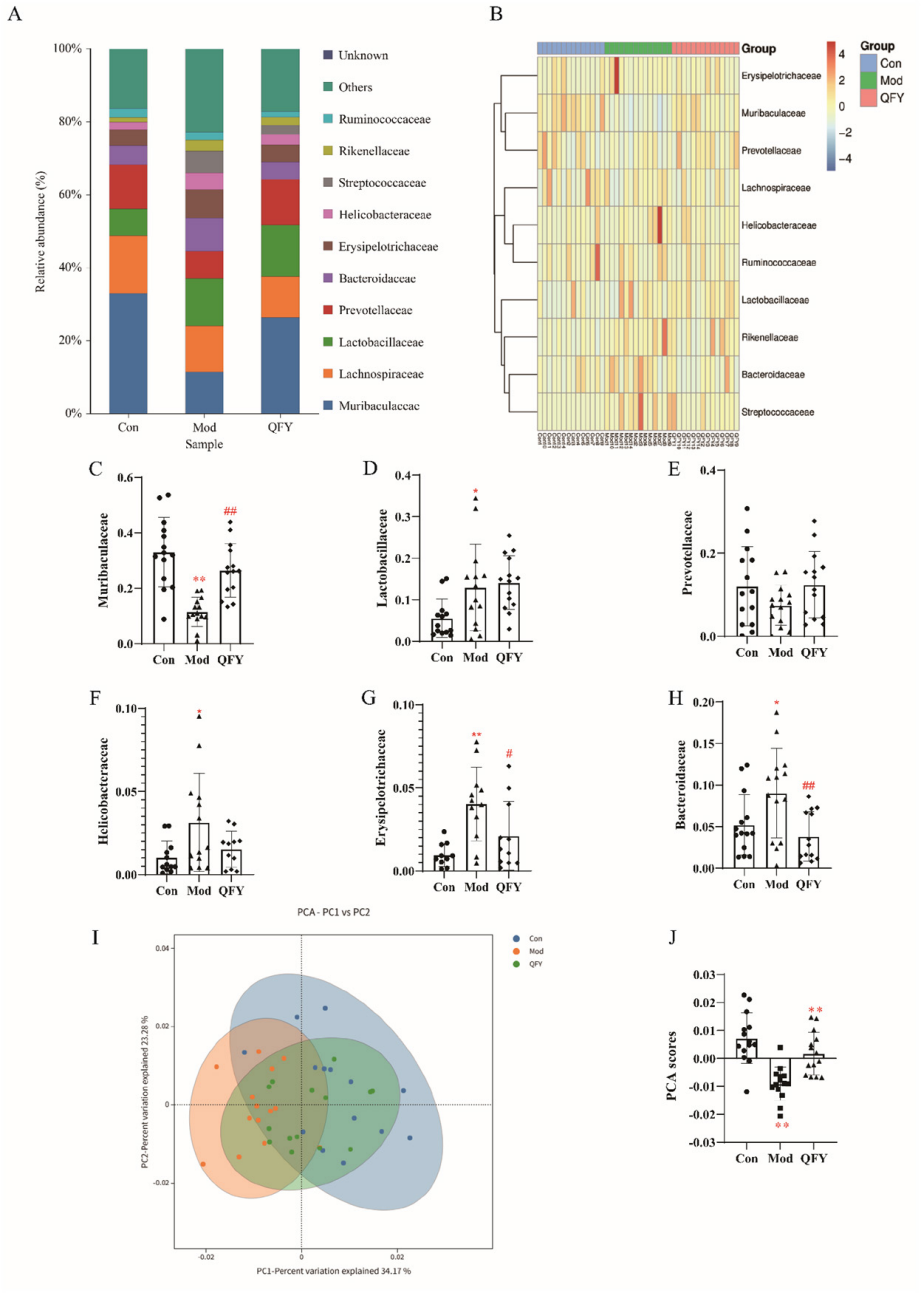
QFY modulates gut microbiota composition and β-diversity at the family level in 3xTg-AD mice. **(A)** Relative abundance of gut microbiota at the family level in Con, Mod, and QFY groups. The stacked bar plot illustrates the proportion of different bacterial families, with colors representing various bacterial families. **(B)** Heatmap of the relative abundance of gut microbiota at the family level. The samples are clustered based on their microbial profiles, with distinct groupings for Con, Mod, and QFY samples. **(C)** Relative abundance of Muribaculaceae in the Con, Mod, and QFY groups. **(D)** Relative abundance of Lachnospiraceae in the Con, Mod, and QFY groups. **(E)** Relative abundance of Lactobacillaceae in the Con, Mod, and QFY groups. **(F)** Relative abundance of Prevotellaceae in the Con, Mod, and QFY groups. **(G)** Relative abundance of Helicobacteraceae in the Con, Mod, and QFY groups. **(H)** Relative abundance of Erysipelotrichaceae in the Con, Mod, and QFY groups. **(I)** Relative abundance of Bacteroidaceae in the Con, Mod, and QFY groups. **(J)** PCA plot illustrating the variation of gut microbiota profiles among the Con (green), Mod (blue), and QFY (orange) groups. The percentage of variance explained by the first two principal components (PC1 and PC2) is shown. **(K)** PCA scores for the Con, Mod, and QFY groups, with statistical significance indicated between groups. *P < 0.05, **P < 0.01 vs. Con, ^#^*P* < 0.05, ^##^*P* < 0.01, vs. Mod, Student‘s *t*-test.

At the family level, the top ten dominant taxa across the three groups were Muribaculaceae, Lachnospiraceae, Lactobacillaceae, Prevotellaceae, Bacteroidaceae, Erysipelotrichaceae, Helicobacteraceae, Streptococcaceae, Rikenellaceae, and Ruminococcaceae (Figure 5A). The hierarchical clustering heatmap further revealed inter-individual variations in the composition of dominant taxa (Figure 5B). At the family level, compared to the Con group, the Mod group exhibited significantly reduced levels of Muribaculaceae (Figure 5C, *P* < 0.01), while Lactobacillaceae (Figure 5D, *P* < 0.05), Helicobacteraceae (Figure 5F, *P* < 0.05), Erysipelotrichaceae (Figure 5G, *P* < 0.01), Bacteroidaceae (Figure 5H, *P* < 0.05) levels were significantly increased. Notably, following QFY treatment, the levels of Muribaculaceae (Figure 5C, *P* < 0.01) were significantly increased, whereas Erysipelotrichaceae (Figure 5C, *P* < 0.05) and Bacteroidaceae (Figure 5H, *P* < 0.01) were significantly decreased. However, no significant differences were observed in Prevotellaceae levels between the Mod and Con groups, nor between the QFY treatment group and the model group (Figure 5E). Pearson correlation analysis revealed that Muribaculaceae showed a significant positive correlation with grip strength normalized to body weight and latency to fall, whereas Bacteroidaceae exhibited a significant negative correlation with grip strength normalized to body weight (Table 1). These findings indicate that QFY effectively modulates the gut microbiota composition at the family level in 3xTg-AD mice. Principal component analysis (PCA) (Figure 5I) revealed that the β-diversity of the QFY group was similar to that of the Con group, whereas the Mod group exhibited significantly lower β-diversity compared to both Con and QFY groups. Statistical analysis of PCA scores revealed that, compared with the Con group, the Mod group exhibited a significant reduction in scores, whereas the QFY group showed a significant increase (Figure 5J).

**TABLE 1:** Correlation between microbial species abundance and frailty phenotypes in 3xTg-AD mice.

Name	Grip strength/weight in grip strength test	Latency to fall in rotarod test
Muribaculaceae	*r* = 0.6189 *P* < 0.0001**	*r* = 0.5045 *P* = 0.0007**
Lactobacillaceae	*r* = −0.1232 *P* = 0.4372	*r* = −0.2035 *P* = 0.1961
Prevotellaceae	*r* = 0.3003 *P* = 0.0533	*r* = 0.07879 *P* = 0.6199
Helicobacteraceae	*r* = −0.2225 *P* = 0.1566	*r* = −0.2218 *P* = 0.1581
Erysipelotrichaceae	*r* = −0.2368 *P* = 0.1310	*r* = −0.1424 *P* = 0.3684
Bacteroidaceae	*r* = −0.3604 *P* = 0.0190*	*r* = −0.1278 *P* = 0.4198

*P < 0.05, **P < 0.01, Pearson correlation analysis, Graphpad 8.0.1.

### 3.6 QFY modulates kEGG-associated gut microbial functions in 3xTg-AD mice

**FIGURE 6:**
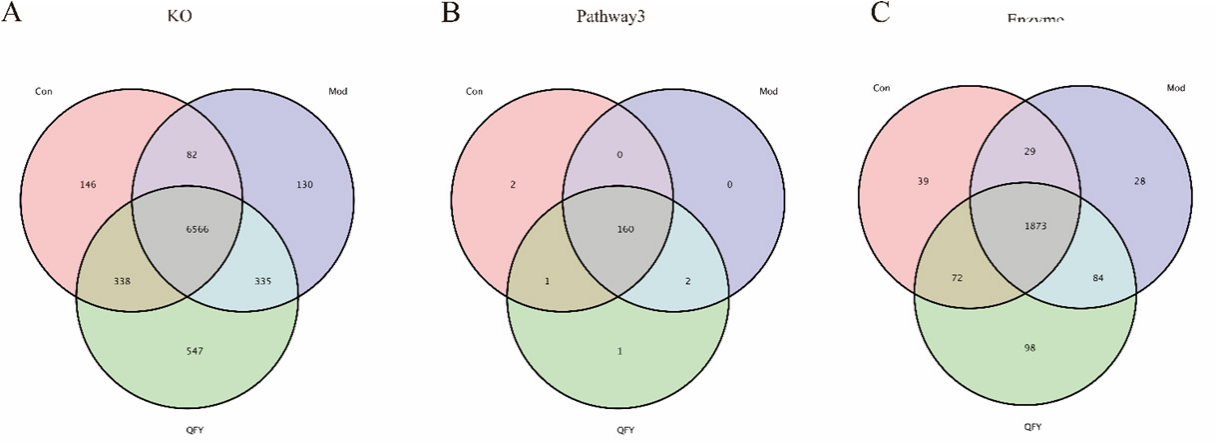
The effect of QFY on the function of intestinal microorganisms in 3xTg-AD mice based on KEGG analysis. **(A)** Venn diagram at the KeggKO level. **(B)** Venn diagram at the KeggPathway3 level. **(C)** Venn diagram at the KeggEnzyme level.

We analyzed the KO, KeggPathway3, and enzyme levels using the KEGG database (Figure 6). In this study, KeggPathway1, KeggPathway2, and KeggPathway3 refer to KEGG’s hierarchical classification of functional pathways, from broad categories (Level 1) to subcategories (Level 2) and specific metabolic or signaling pathways (Level 3), respectively. The results showed that at the KO level, the Con group and the Mod group shared 82 functions, while the Con group shared 338 functions with the QFY group (Figure 6A). At the KeggPathway3 level, the Con and Mod groups had no shared functions, but the Con group shared one function with the QFY group (Figure 6B). At the enzyme level, the Con and Mod groups shared 29 functions, whereas the Con group shared 72 functions with the QFY group (Figure 6C).

Further analysis of detailed functions at the KeggPathway1, KeggPathway2, KeggPathway3, KO, and enzyme levels revealed that, at the KeggPathway1 level, the environmental information processing function was significantly higher in the Mod group compared to the Con group, while the genetic information processing and metabolism functions were significantly lower. In the QFY group, the environmental information processing function was significantly reduced compared to the Mod group, while the genetic information processing function was significantly increased (Table 2). Pearson correlation analysis indicated that environmental information processing was significantly negatively correlated with latency to fall, Metabolism was significantly positively correlated with both grip strength normalized to body weight and latency to fall, and Cellular Processes showed significant negative correlations with grip strength normalized to body weight and latency to fall (Table 3).

**TABLE 2:** Gene expression analysis of intestinal microorganisms in 3xTg-AD mice treated by QFY in KEGG database.

KEGG level	Name	Proportion% (mean ± SD)		
		Con	Mod	QFY
KeggPathway1	Environmental information processing	0.1130 ± 0.0218	0.1394 ± 0.0282↑	0.1170 ± 0.0189↓
	Genetic information processing	0.2292 ± 0.0090	0.2187 ± 0.0132↓	0.2306 ± 0.0081↑↑
	Metabolism	0.6074 ± 0.0324	0.5720 ± 0.0441↓	0.5986 ± 0.0274
	Cellular processes	0.0513 ± 0.0105	0.06699 ± 0.0234	0.0538 ± 0.0132
KeggPathway2	Carbohydrate metabolism	0.1060 ± 0.0037	0.1065 ± 0.0041	0.1064 ± 0.0020
	Global and overview maps	0.3117 ± 0.0116	0.2998 ± 0.0143↓	0.3098 ± 0.0093↑
	Amino acid metabolism	0.0858 ± 0.0036	0.0807 ± 0.0049↓	0.0833 ± 0.0024
	Translation	0.0633 ± 0.0044	0.0626 ± 0.0044	0.0652 ± 0.0030
	Metabolism of cofactors and vitamins	0.0635 ± 0.0045	0.0583 ± 0.0050↓↓	0.0620 ± 0.0044↑
	Nucleotide metabolism	0.0573 ± 0.0018	0.0557 ± 0.0020↓	0.0595 ± 0.0013↑↑
	Membrane transport	0.0442 ± 0.0079	0.0564 ± 0.0112↑↑	0.0472 ± 0.0074↓
	Replication and repair	0.0389 ± 0.0020	0.0374 ± 0.0022	0.0397 ± 0.0007↑↑
KeggPathway3	Metabolic pathways	0.1946 ± 0.0067	0.1866 ± 0.0081↓↓	0.1933 ± 0.0057↑
	Microbial metabolism in diverse environments	0.0409 ± 0.0010	0.0425 ± 0.0025↑	0.0417 ± 0.0011
	Purine metabolism	0.0252 ± 0.0010	0.0251 ± 0.0007	0.0262 ± 0.0004↓↓
	Pyrimidine metabolism	0.0222 ± 0.0007	0.0212 ± 0.0008↓↓	0.0231 ± 0.0006↑↑
	ABC transporters	0.0190 ± 0.0043	0.0241 ± 0.0053↑↑	0.0199 ± 0.0037↓
KO	K03088	0.0120 ± 0.0010	0.0091 ± 0.0031↓↓	0.0100 ± 0.0014
	K02004	0.0072 ± 0.0013	0.0052 ± 0.0010↓↓	0.0060 ± 0.0016
	K01990	0.0043 ± 0.0016	0.0051 ± 0.0019	0.0045 ± 0.0014
	K01992	0.0037 ± 0.0007	0.0040 ± 0.0010	0.0039 ± 0.0005
Enzyme	EC:3.6.4.12	0.0198 ± 0.0013	0.0172 ± 0.0015↓↓	0.0193 ± 0.0011↑↑
	EC:2.7.7.7	0.0150 ± 0.0017	0.0151 ± 0.0017	0.0156 ± 0.0008
	EC:3.1.-.-	0.0134 ± 0.0011	0.0146 ± 0.0016	0.0135 ± 0.0008
	EC:3.1.21.3	0.0105 ± 0.0016	0.0093 ± 0.0013↓	0.0107 ± 0.0015↑
	EC:1.6.5.3	0.0102 ± 0.0023	0.0069 ± 0.0019↓↓	0.0105 ± 0.0023↑↑
	EC:3.4.24.-	0.0088 ± 0.0004	0.0083 ± 0.0007↓	0.0090 ± 0.0004↑↑
	EC:5.2.1.8	0.0094 ± 0.0014	0.0074 ± 0.0019↓↓	0.0089 ± 0.0013↑

↑ and ↓ means *P* < 0.05, ↑↑ and ↓↓ means *P* < 0.01.

**TABLE 3:** Correlation between gene expression of KEGG database and frailty phenotypes in 3xTg-AD mice.

Name	Grip strength/weight in grip strength test	Latency to fall in rotarod test
Environmental information processing	*r* = 0.0251 *P* = 0.8746	*r* = −0.4558 *P* = 0.0024**
Genetic information processing	*r* = 0.2693 *P* = 0.0846	*r* = −0.2044 *P* = 0.1941
Metabolism	*r* = 0.4399 *P* = 0.0036*	*r* = 0.3901 *P* = 0.0106*
Cellular processes	*r* = −0.4071 *P* = 0.0075**	*r* = −0.3217 *P* = 0.0378*
Carbohydrate metabolism	*r* = 0.1801 *P* = 0.2536	*r* = −0.0070 *P* = 0.9651
Global and overview maps	*r* = −0.0518 *P* = 0.7446	*r* = −0.0379 *P* = 0.8116
Amino acid metabolism	*r* = −0.0276 *P* = 0.8621	*r* = 0.01433 *P* = 0.9282
Translation	*r* = −0.1304 *P* = 0.4103	*r* = −0.0475 *P* = 0.7652
Metabolism of cofactors and vitamins	*r* = −0.1192 *P* = 0.4522	*r* = −0.0606 *P* = 0.7030
Nucleotide metabolism	*r* = −0.2384 *P* = 0.1284	*r* = −0.2024 *P* = 0.1986
Membrane transport	*r* = 0.1044 *P* = 0.5107	*r* = 0.0510 *P* = 0.7486
Replication and repair	*r* = −0.2721 *P* = 0.0813	*r* = −0.2154 *P* = 0.1708
Metabolic pathways	*r* = 0.5101 *P* = 0.0006**	*r* = 0.4767 *P* = 0.0014*
Microbial metabolism in diverse environments	*r* = −0.2827 *P* = 0.0697	*r* = −0.2958 *P* = 0.0572
Purine metabolism	*r* = 0.1801 *P* = 0.2537	*r* = 0.0318 *P* = 0.8417
Pyrimidine metabolism	*r* = 0.3388 *P* = 0.0282*	*r* = 0.2326 *P* = 0.1382
ABC transporters	*r* = −0.4681 *P* = 0.0018**	*r* = −0.4044 *P* = 0.0079**
K03088	*r* = 0.3646 *P* = 0.0176*	*r* = 0.5161 *P* = 0.0005**
K02004	*r* = 0.1055 *P* = 0.5061	*r* = 0.2772 *P* = 0.0755
K01990	*r* = −0.2255 *P* = 0.1510	*r* = −0.0774 *P* = 0.6260
K01992	*r* = −0.1769 *P* = 0.2624	*r* = −0.07736 *P* = 0.6263
EC:3.6.4.12	*r* = 0.5377 *P* = 0.0002**	*r* = 0.5223 *P* = 0.0004**
EC:2.7.7.7	*r* = −0.1375 *P* = 0.3851	*r* = −0.1808 *P* = 0.2518
EC:3.1.-.-	*r* = −0.3498 *P* = 0.0232*	*r* = −0.3049 *P* = 0.0496*
EC:3.1.21.3	*r* = 0.2380 *P* = 0.1291	*r* = 0.4025 *P* = 0.0082**
EC:1.6.5.3	*r* = 0.5002 *P* = 0.0007**	*r* = 0.4096 *P* = 0.0071**
EC:3.4.24.-	*r* = −0.5020 *P* = 0.0007**	*r* = 0.3758 *P* = 0.0142*
EC:5.2.1.8	*r* = 0.5098 *P* = 0.0006**	*r* = 0.4092 *P* = 0.0071**

*P < 0.05, **P < 0.01, Pearson correlation analysis, Graphpad 8.0.1.

At the KeggPathway2 level, compared to the Con group, the Mod group showed significant decreases in the Global and Overview Maps, Amino Acid Metabolism, Metabolism of Cofactors and Vitamins, and Nucleotide Metabolism functions, but a significant increase in Membrane Transport. Compared to the Mod group, the QFY group showed significant increases in the Global and Overview Maps, Metabolism of Cofactors and Vitamins, Nucleotide Metabolism, and Replication and Repair functions, but a significant decrease in Membrane Transport (Table 2).

At the KeggPathway3 level, compared to the Con group, the Mod group exhibited significant reductions in Metabolic Pathways and Pyrimidine Metabolism, but significant increases in Microbial Metabolism in Diverse Environments and ABC Transporters. In contrast, compared to the Mod group, the QFY group showed significant increases in Metabolic Pathways and Pyrimidine Metabolism, while Purine Metabolism and ABC Transporters were significantly reduced (Table 2). Pearson correlation analysis showed that Metabolic Pathways were significantly positively correlated with grip strength normalized to body weight and latency to fall, whereas Pyrimidine metabolism exhibited a significant negative correlation with grip strength normalized to body weight (Table 3).

At the KO level, compared to the Con group, the Mod group showed significant reductions in the K03088 and K02004 functions (Table 2). Pearson correlation analysis revealed that K03088 was significantly positively correlated with grip strength normalized to body weight and latency to fall (Table 3).

At the enzyme level, compared to the Con group, the Mod group showed significant reductions in EC:3.6.4.12, EC:3.1.21.3, EC:1.6.5.3, EC:3.4.24.-, and EC:5.2.1.8. Compared to the Mod group, the QFY group showed significant increases in these enzyme functions (Table 2). Pearson correlation analysis showed that EC:3.6.4.12, EC:1.6.5.3, and EC:5.2.1.8 were significantly positively correlated with grip strength normalized to body weight and latency to fall, whereas EC:3.1.-.- exhibited a significant negative correlation with both indicators. In addition, EC:3.1.21.3 was significantly positively correlated with latency to fall (Table 3).

### 3.7 QFY modulates fecal eggNOG-associated functional genes in 3xTg-AD mice

**FIGURE 7:**
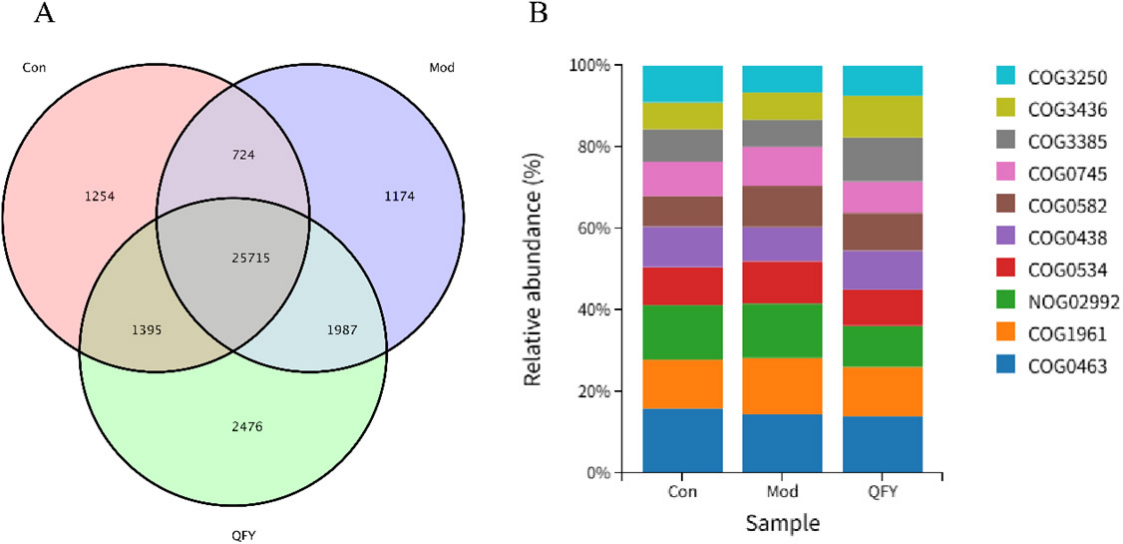
The effect of QFY on the function of eggNOG of intestinal microorganisms in 3xTg-AD mice. **(A)** Overview of functional profiles at the EggNOG NOG level across groups. **(B)** Top 10 community abundance percentages at the EggNOG NOG functional level across groups.

Analysis of eggNOG-related functional genes in the feces of mice revealed that the Con group and the Mod group shared 724 functions, while the Con group shared 1,395 functions with the QFY group (Figure 7A). The top ten most abundant functional genes across the three groups were: COG0463, COG1916, NOG02992, COG0534, COG0438, COG0582, COG0745, COG3385, COG3436, COG3250 (Figure 7B).

Functional annotation of the gut microbiota was conducted using the eggNOG database to investigate potential alterations in microbial functions among groups. At the NOG level, several functional orthologs showed significant variations between the Mod and Con mice. Specifically, COG0438, associated with ribosomal protein synthesis, was markedly decreased in the Mod group (Table 4, *P* < 0.01) but partially restored following the treatment of QFY (Table 4, *P* < 0.05). In contrast, COG3385 and COG3436 were significantly increased after administration of QFY (Table 4, *P* < 0.01), pearson correlation analysis showed that COG0438 and COG3385 were significantly positively correlated with grip strength normalized to body weight and latency to fall (Table 5), suggesting that QFY may enhance microbial functions related to signal transduction and secondary metabolite biosynthesis. At the functional class level, the “[M]: Cell wall/membrane/envelope biogenesis” class was significantly reduced in the Mod group (Table 4, *P* < 0.05) but showed a tendency toward recovery after QFY treatment, though not to a statistically significant extent, pearson correlation analysis showed that [M]: Cell wall/membrane/envelope biogenesis were significantly positively correlated with grip strength normalized to body weight and latency to fall (Table 5). This finding indicates that QFY may partly restore bacterial structural integrity that was compromised in the AD Mod.

**TABLE 4:** Gene expression analysis of intestinal microorganisms in 3xTg-AD mice treated by QFY in eggNOG database.

level	Name	Proportion% (mean ± SD)		
		Con	Mod	QFY
NOG	COG0463	0.0056 ± 0.0004	0.0049 ± 0.0010↓	0.0049 ± 0.0004
	COG1961	0.0042 ± 0.0022	0.0034 ± 0.0030	0.0042 ± 0.0027
	NOG2992	0.0030 ± 0.0007	0.0045 ± 0.0056	0.0018 ± 0.0005
	COG0438	0.0036 ± 0.0004	0.0029 ± 0.0007↓↓	0.0034 ± 0.0004↑
	COG0534	0.0033 ± 0.0006	0.0036 ± 0.0008	0.0031 ± 0.0008
	COG0745	0.0029 ± 0.0010	0.0033 ± 0.0012	0.0027 ± 0.0010
	COG0582	0.0026 ± 0.0008	0.0034 ± 0.0009↑	0.0032 ± 0.0011
	COG3385	0.0028 ± 0.0008	0.0022 ± 0.0011	0.0038 ± 0.0014↑↑
	COG3436	0.0023 ± 0.0009	0.0021 ± 0.0005	0.0035 ± 0.0016↑↑
Category	POORLY CHARACTERIZED	0.3532 ± 0.0155	0.3412 ± 0.0219	0.3418 ± 0.0157
	METABOLISM	0.2499 ± 0.0177	0.2542 ± 0.0264	0.2534 ± 0.0196
	INFORMATION STORAGE AND PROCESSING	0.1998 ± 0.0094	0.2075 ± 0.0054↑	0.2148 ± 0.0087↑
	CELLULAR PROCESSES AND SIGNALING	0.1971 ± 0.0063	0.1949 ± 0.0105	0.1899 ± 0.0067
Class	[L]: Replication, recombination and repair	0.0930 ± 0.0057	0.0977 ± 0.0045↑	0.1059 ± 0.0085↑↑
	[R]: General function Prediction only	0.0818 ± 0.0015	0.0832 ± 0.0047	0.0790 ± 0.0018↓↓
	[M]: Cell wall/membrane/envelope biogenesis	0.0730 ± 0.0055	0.0676 ± 0.0066↓	0.0710 ± 0.0052
	[G]: Carbohydrate transport and metabolism	0.0598 ± 0.0026	0.0601 ± 0.0053	0.0597 ± 0.0033
	[J]: Translation, ribosomal structure and biogenesis	0.0578 ± 0.0057	0.0593 ± 0.0089	0.0603 ± 0.0057
	[E]: Amino acid transport and metabolism	0.0504 ± 0.0045	0.0528 ± 0.0061	0.0514 ± 0.0046
	[K]: Transcription	0.0474 ± 0.0039	0.0506 ± 0.0060	0.0471 ± 0.0045
	[C]: Energy production and conversion	0.0376 ± 0.0040	0.0384 ± 0.0050	0.0383 ± 0.0029
	[P]: Inorganic ion transport and metabolism	0.0368 ± 0.0032	0.0375 ± 0.0049	0.0375 ± 0.0037

↑ and ↓ means *P* < 0.05, ↑↑ and ↓↓ means *P* < 0.01.

**TABLE 5:** Correlation between gene expression of eggNOG database and frailty phenotypes in 3xTg-AD mice.

Name	Grip strength/ Weight in grip strength test	Latency to fall in rotarod test
COG0463	*r* = 0.0251 *P* = 0.8746	*r* = 0.1425 *P* = 0.3681
COG1961	*r* = −0.1973 *P* = 0.2105	*r* = −0.1937 *P* = 0.2190
NOG2992	*r* = 0.0273 *P* = 0.8636	*r* = 0.0462 *P* = 0.7713
COG0438	*r* = 0.4458 *P* = 0.0031**	*r* = 0.4547 *P* = 0.0025**
COG0745	*r* = −0.2645 *P* = 0.0905	*r* = −0.1171 *P* = 0.4602
COG0582	*r* = −0.2329 *P* = 0.1377	*r* = −0.1663 *P* = 0.2924
COG3385	*r* = 0.4807 *P* = 0.0013**	*r* = 0.4061 *P* = 0.0076**
COG3436	*r* = 0.2316 *P* = 0.1400	*r* = 0.2955 *P* = 0.0574
POORLY CHARACTERIZED	*r* = 0.1514 *P* = 0.3386	*r* = 0.2961 *P* = 0.0569
METABOLISM	*r* = −0.0014 *P* = 0.9931	*r* = −0.1185 *P* = 0.4548
INFORMATION STORAGE AND PROCESSING	*r* = −0.1119 *P* = 0.4806	*r* = −0.1644 *P* = 0.2981
CELLULAR PROCESSES AND SIGNALING	*r* = −0.1792 *P* = 0.2561	*r* = −0.1316 *P* = 0.4061
[L]: Replication, recombination and repair	*r* = 0.1086 *P* = 0.4937	*r* = 0.0701 *P* = 0.6594
[R]: General function Prediction only	*r* = −0.08834 *P* = 0.5780	*r* = −0.0104 *P* = 0.9478
[M]: Cell wall/membrane/envelope biogenesis	*r* = 0.3449 *P* = 0.0253*	*r* = 0.3120 *P* = 0.0443*
[G]: Carbohydrate transport and metabolism	*r* = 0.05492 *P* = 0.7298	*r* = −0.0643 *P* = 0.6859
[J]: Translation, ribosomal structure and biogenesis	*r* = −0.0215 *P* = 0.8924	*r* = −0.1331 *P* = 0.4007
[E]: Amino acid transport and metabolism	*r* = −0.1295 *P* = 0.4136	*r* = −0.2769 *P* = 0.0759
[K]: Transcription	*r* = −0.3591 *P* = 0.0195*	*r* = −0.2554 *P* = 0.1026
[C]: Energy production and conversion	*r* = −0.0547 *P* = 0.7308	*r* = −0.1175 *P* = 0.4585
[P]: Inorganic ion transport and metabolism	*r* = 0.03302 *P* = 0.8355	*r* = −0.0149 *P* = 0.9252

*P < 0.05, **P < 0.01, Pearson correlation analysis, Graphpad 8.0.1.

### 3.8 QFY regulates fecal carbohydrate-active enzymes and their correlation with motor performance in 3xTg-AD mice

**FIGURE 8:**
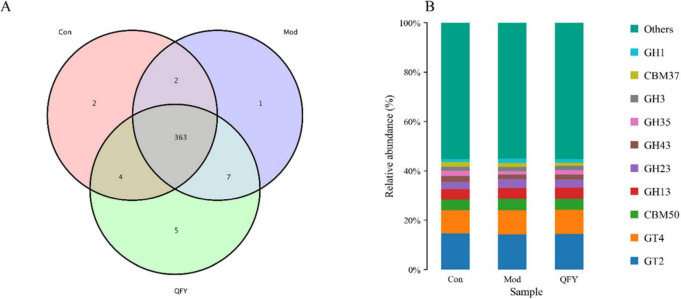
The effect of QFY on CAZyme of intestinal microorganisms in 3xTg-AD mice. **(A)** Overview of functional profiles at the CAZyme level across groups. **(B)** Relative abundance of major CAZyme families in the Con, Mod, and QFY groups. The stacked bar plots indicate the proportional distribution of different enzyme families, including glycoside hydrolases (GHs), carbohydrate-binding modules (CBMs), and glycosyltransferases (GTs).

The analysis of carbohydrate-active enzymes revealed that the Con group and the Mod group shared 2 enzymes, while the Con group shared 4 enzymes with the QFY group (Figure 8A). The top ten most abundant enzymes across the three groups were: GT2, GT4, CBM50, GH13, GH23, GH43, GH35, GH3, CBM37, GH1 (Figure 8B). Compared with the Con group, the Mod group exhibited significant downregulation of GH43, GH35, GH5, and GH16, while GH73, GH23, GH2, GH1, GH25, GT51, GH20, GH38, and CBM50 were significantly upregulated. Treatment with QFY markedly increased the abundance of GH43, GH2 and GH51, while significantly reducing the abundance of GH73, GH5 and CBM50 (Table 6).

**TABLE 6:** Gene expression analysis of intestinal microorganisms in 3xTg-AD mice treated by QFY in CAZy database.

Name	Con	Mod	QFY
	Proportion% (mean ± SD)		
GH43	0.0235 ± 0.0034	0.0177 ± 0.0031↓↓	0.0201 ± 0.0024↑
GH73	0.0084 ± 0.0018	0.0119 ± 0.004↑↑	0.0094 ± 0.0019↓
GH23	0.0304 ± 0.0037	0.0373 ± 0.0055↑↑	0.0342 ± 0.0031
GH35	0.0207 ± 0.0063	0.0137 ± 0.0075↓	0.0177 ± 0.0049
GH2	0.0161 ± 0.0024	0.0135 ± 0.0028↑	0.0146 ± 0.0015↑↑
GH5	0.0148 ± 0.0016	0.0132 ± 0.0022↓	0.0140 ± 0.0017↓
GH1	0.0131 ± 0.0030	0.0177 ± 0.0057↑	0.0161 ± 0.0033
GH25	0.0118 ± 0.0023	0.0151 ± 0.0037↑↑	0.0134 ± 0.0019
GT51	0.0124 ± 0.0021	0.0145 ± 0.0029↑↑	0.0130 ± 0.0013↑
GH16	0.0122 ± 0.0027	0.0095 ± 0.0035↓	0.0107 ± 0.0022
GH20	0.0085 ± 0.0006	0.0101 ± 0.0020↑	0.0090 ± 0.0010
GH38	0.0082 ± 0.0015	0.0095 ± 0.0018↑	0.0082 ± 0.0017
CBM50	0.0429 ± 0.0030	0.0475 ± 0.0037↑↑	0.0452 ± 0.0021↓

↑ and ↓ means *P* < 0.05, ↑↑ and ↓↓ means *P* < 0.01.

Pearson correlation analysis between the abundance of carbohydrate-active enzymes and behavioral performance (grip strength/body weight ratio and time on the rotarod) showed that CBM50, GH73, GH23, GH25, and GH38 abundances were significantly negatively correlated with holding power/body weight ratio, whereas GH43, GH35, GH2, and GH5 abundances were significantly positively correlated. Similarly, CBM50, GH73, GH23, GH1, GH25, GT51, and GH20 abundances were significantly negatively correlated with rotarod time, while GH43 and GH35 abundances were significantly positively correlated (Table 7).

**TABLE 7:** Correlation between gene expression of CAZy database and frailty phenotypes in 3xTg-AD mice.

Name	Grip strength/ weight in grip strength test	Latency to fall in rotarod test
GH43	*r* = 0.3340 *P* = 0.0306*	*r* = 0.3219 *P* = 0.0376*
GH73	r = −0.4172 *P* = 0.0060**	*r* = −0.4202 *P* = 0.0056**
GH23	*r* = −0.3496 *P* = 0.0232*	*r* = −0.4345 *P* = 0.0040**
GH35	*r* = 0.3056 *P* = 0.0490*	*r* = 0.3287 *P* = 0.0336*
GH2	*r* = 0.3649 *P* = 0.0175*	*r* = 0.2453 *P* = 0.1174
GH5	*r* = 0.3565 *P* = 0.0205*	*r* = 0.1392 *P* = 0.3793
GH1	*r* = −0.3028 *P* = 0.0512	*r* = −0.4123 *P* = 0.0067**
GH25	*r* = −0.3656 *P* = 0.0173*	*r* = −0.3839 *P* = 0.0121*
GT51	*r* = −0.3029 *P* = 0.0512	*r* = −0.3191 *P* = 0.0395*
GH16	*r* = 0.2743 *P* = 0.0788	*r* = 0.2975 *P* = 0.0557
GH20	*r* = −0.1956 *P* = 0.2144	*r* = −0.3373 *P* = 0.0289*
GH38	*r* = −0.3358 *P* = 0.0297*	*r* = −0.2774 *P* = 0.0753
CBM50	*r* = −0.4126 *P* = 0.0066**	*r* = −0.3522 *P* = 0.0222*

*P < 0.05, **P < 0.01, Pearson correlation analysis, Graphpad 8.0.1.

Based on the above data, results demonstrated that (i) gut microbiota dysbiosis occurs in 3xTg-AD mice at both taxonomic and functional levels; (ii) QFY treatment effectively reshaped the microbial composition, restoring it toward the pattern of the Con group; (iii) QFY also improved functional features of the microbiome, particularly in pathways related to metabolism, genetic information processing, and carbohydrate enzyme activity.

## 4 Discussion

Our previous work showed that QFY improved limb strength, motor coordination, and cold stress resistance in APP/PS1 mice (Xiao et al., 2022). Building upon this foundation, the present study employed 3xTg-AD mice, which harbor APP, PS1, and MAPT mutations, and expanded the behavioral assessments by adding aging score assessment or frailty scoring in addition to grip strength and rotarod tests. Consistently, QFY significantly improved limb strength and motor coordination in 3xTg-AD mice and delayed age-related functional decline. It should be acknowledged that aging itself may represent an important confounding factor when interpreting these behavioral outcomes. In the 3xTg-AD model, Aβ and tau pathologies begin to emerge as early as 6 months of age and progressively worsen with aging, whereas pronounced peripheral functional impairments—such as skeletal muscle bioenergetic dysfunction—tend to become evident around 12 months of age (Belfiore et al., 2019). Moreover, age-associated functional decline, including gait impairment and frailty-related features, has been shown to intensify with increasing age in this model (Castillo-Mariqueo et al., 2021). Therefore, part of the observed deterioration in motor performance and physiological function during the experimental period may reflect the natural trajectory of aging rather than disease-specific effects alone. This age-related progression should be taken into account when interpreting both baseline functional decline and the magnitude of intervention-associated improvements observed in long-term studies. Regarding microbial composition, earlier findings suggested that QFY increased the abundance of Bacteroidaceae (Xiao et al., 2022). In contrast, the current study revealed that QFY reduced the elevated levels of Bacteroidaceae observed in the AD model group, this discrepancy may stem from differences in disease models, baseline microbiota structures, and pathological states. Of particular interest, Muribaculaceae—widely recognized as a beneficial taxon in maintaining gut health (He et al., 2024; Xia et al., 2023; Jiao et al., 2025), was significantly restored following QFY treatment. Its positive correlations with grip strength and motor coordination suggest a close association between Muribaculaceae abundance and physical performance. However, this study also revealed some differences from prior observations. Specifically, we found significant elevations of Alcaligenaceae, Rhodanobacteraceae, and Spirochaetaceae in the Mod group, all of which decreased after QFY treatment. Although these taxa have been associated with inflammation and metabolic dysfunction in certain contexts, their biological implications may vary across disease models (Hu et al., 2024; Xiao et al., 2024; Agranyoni et al., 2024). For example, increased Alcaligenaceae abundance has been reported in inflammatory bowel disease models (Shi et al., 2021); Rhodanobacteraceae, though uncommon in the gut, may reflect specific inflammatory or environmental stress responses (Modlin et al., 2021); Spirochaetaceae has been linked to intestinal inflammation and barrier impairment (Wood et al., 2022). Thus, QFY-mediated reductions in these taxa may be associated with a less inflammatory gut environment and improved barrier-related microbial features, which are commonly linked to frailty in AD. Supporting this, gut dysbiosis is known to increase intestinal permeability and systemic inflammation, leading to downstream effects on neural function (Agranyoni et al., 2024). Moreover, β-diversity analysis showed that QFY restored the overall microbial community structure of 3xTg-AD mice, rendering it similar to healthy controls. Growing evidence supports a strong relationship between gut microbiota and physical frailty (Yin et al., 2025; Wen et al., 2024). For instance, the dietary gut microbiome index (DI-GM) has been linked to frailty in older adults, with inflammatory markers acting as mediators (Yin et al., 2025). In this study, the positive correlations between Muribaculaceae and both grip strength and motor coordination, together with the negative correlation between Bacteroidaceae and normalized grip strength, provide supportive associative evidence for microbiota–frailty interactions. Bacteroidaceae, a dominant gut family, has been associated with various pathological conditions—such as alterations induced by high-fat diets leading to obesity and metabolic disorders (Wu et al., 2023; Gomes et al., 2021). Its negative association with grip strength in this study suggests a potential detrimental role in AD-related frailty.

It should also be noted that the positive drug group in this study consisted of a combination of donepezil and memantine, which are widely used as standard pharmacological treatments for cognitive symptoms in Alzheimer’s disease. These agents were included to provide a clinically relevant reference for AD treatment rather than as established positive controls for motor frailty or gut microbiota modulation. To date, there is limited evidence supporting the efficacy of donepezil or memantine in reversing frailty-related motor deficits or reshaping gut microbial composition, particularly in 3xTg-AD mice.

Therefore, the comparative analyses in the present study were not intended to evaluate the superiority of QFY over conventional cognitive drugs. Instead, they were designed to highlight the distinct phenotypic domains targeted by QFY, especially motor performance and gut microbiota remodeling, which are not primary therapeutic targets of donepezil or memantine. From this perspective, QFY may offer complementary benefits beyond standard AD pharmacotherapy, particularly in addressing frailty-related and microbiota-associated alterations.

Another major contribution of this study lies in revealing the effects of QFY on microbial functional genes and metabolic pathways. It should be noted that functional annotations based on KEGG, eggNOG, and CAZy analyses reflect predicted microbial functional potential inferred from sequencing data, rather than direct biochemical activity or metabolite concentrations. KEGG functional annotation demonstrated that QFY reversed the model-induced increase in environmental information processing and restored decreases in genetic information processing and metabolic functions. Importantly, metabolic pathways were positively correlated with grip strength and latency to fall, suggesting that QFY is associated with enhanced microbial metabolic potential, which may favor the production of beneficial metabolites. Short-chain fatty acids (SCFAs), for example, derived from microbial fermentation of dietary fibers, play essential roles in maintaining barrier integrity, regulating immunity, and influencing neural function (Xu et al., 2020; Xia et al., 2023). QFY may be associated with alterations in microbial pathways related to SCFA production—potentially involving Muribaculaceae or other beneficial taxa—which have been widely associated with frailty-related and neurodegenerative phenotypes in previous studies. Consistent with this interpretation, previous studies have shown that modulating gut microbiota and microbial metabolites can ameliorate cognitive impairment in AD models (Wang et al., 2025; Li et al., 2025; Yang et al., 2025).

Functional gene analysis using the eggNOG database further supported QFY-mediated regulatory effects on gut microbiota. QFY restored the decreased abundance of COG0438 (ribosomal protein synthesis) in the Mod group and upregulated COG3385 and COG3436 (associated with signal transduction and secondary metabolite biosynthesis). COG0438 and COG3385 were positively correlated with normalized grip strength and latency to fall. Additionally, the “cell wall/membrane/envelope biogenesis” category, which was markedly reduced in the Mod group, showed a trend toward recovery following QFY treatment and was positively associated with physical performance. Given that bacterial cell wall/membrane integrity is crucial for maintaining intestinal barrier function—and that impaired barrier function is a notable feature in AD pathology—these findings suggest that QFY is associated with changes in microbial functional categories related to cell wall and membrane biogenesis, which have been linked to intestinal barrier integrity in previous studies (Gámez-Macías et al., 2024).

Finally, the regulation of fecal carbohydrate-active enzymes (CAZymes) and their associations with motor performance adds an additional functional dimension for understanding QFY’s microbiota-associated effects. CAZymes mediate the degradation and utilization of carbohydrates, thereby shaping microbial metabolic activity and host energy homeostasis. QFY reversed aberrant CAZyme expression observed in the Mod group, increasing the abundance of GH43, which were positively correlated with normalized grip strength and rotarod performance. These results indicate that QFY may enhance the fermentation and processing of complex carbohydrates, subsequently improving host energy metabolism and neuromuscular function (Wang et al., 2024; Zhao et al., 2025).

## 5 Conclusion

In summary, this study demonstrates that QFY exerts significant modulatory effects on gut microbiota composition and functional potential in 3xTg-AD mice, accompanied by improvements in motor performance and attenuation of age-related physiological frailty. QFY treatment was associated with the restoration of beneficial microbial taxa, normalization of dysregulated KeggPathways, enzymes, and functional genes, and the emergence of coherent microbiota–enzyme–behavior associations. These findings reinforce the close link between gut dysbiosis and frailty-related phenotypes in Alzheimer’s disease and provide multi-level evidence supporting the involvement of the microbiota–gut–brain axis in QFY-mediated effects.

Several limitations should be acknowledged. The functional analyses were based on predicted microbial potential derived from metagenomic data, and direct measurements of microbial metabolites, such as short-chain fatty acids, were not performed. In addition, fecal microbiota may not fully capture mucosa-associated microbial communities that interact more directly with host immunity. Finally, although strong associations were identified between microbial features and behavioral outcomes, causal relationships remain to be established and will require targeted microbiota manipulation approaches in future studies.

Overall, these findings highlight the potential of QFY as a complementary intervention targeting gut microbiota–associated frailty and functional decline in AD, and provide a foundation for further mechanistic and translational investigations of traditional Chinese medicine within the context of neurodegenerative diseases.

## Data Availability

The data presented in this study are publicly available. The data can be found here: https://www.ncbi.nlm.nih.gov, accession PRJNA1424173.
